# Identification of the Cytosolic Glucose-6-Phosphate Dehydrogenase Gene from Strawberry Involved in Cold Stress Response

**DOI:** 10.3390/ijms21197322

**Published:** 2020-10-03

**Authors:** Yunting Zhang, Mengwen Luo, Lijuan Cheng, Yuanxiu Lin, Qing Chen, Bo Sun, Xianjie Gu, Yan Wang, Mengyao Li, Ya Luo, Xiaorong Wang, Yong Zhang, Haoru Tang

**Affiliations:** 1 College of Horticulture, Sichuan Agricultural University, Chengdu 611130, China; asyunting@gmail.com (Y.Z.); rokki95@hotmail.com (M.L.); ccchengccchong@163.com (L.C.); linyx@sicau.edu.cn (Y.L.); supnovel@gmail.com (Q.C.); sunadam011@163.com (B.S.); wangyanwxy@163.com (Y.W.); limy@sicau.edu.cn (M.L.); luoya945@163.com (Y.L.); Wangxr@sicau.edu.cn (X.W.); 2 Institute of Pomology and Olericulture, Sichuan Agricultural University, Chengdu 611130, China; 3 Mianyang Academy of Agricultural Sciences, Mianyang 621000, China; heyggu@gmail.com

**Keywords:** glucose-6-phosphate dehydrogenase, cold stress, strawberry, antioxidant enzymes, oxidative damage

## Abstract

Glucose-6-phosphate dehydrogenase (G6PDH) plays an important role in plant stress responses. Here, five *FaG6PDH* sequences were obtained in strawberry, designated as *FaG6PDH-CY*, *FaG6PDH-P1*, *FaG6PDH-P1.1*, *FaG6PDH-P2* and *FaG6PDH-P0*, which were divided into cytosolic (CY) and plastidic (P) isoforms based on the bioinformatic analysis. The respective *FaG6PDH* genes had distinct expression patterns in all tissues and at different stages of fruit development. Notably, *FaG6PDH-CY* was the most highly expressed gene among five *FaG6PDH* members, indicating it encoded the major G6PDH isoform throughout the plant. FaG6PDH positively regulated cold tolerance in strawberry. Inhibition of its activity gave rise to greater cold-induced injury in plant. The *FaG6PDH-CY* transcript had a significant increase under cold stress, similar to the G6PDH enzyme activity, suggesting a principal participant in response to cold stress. Further study showed that the low-temperature responsiveness (LTR) element in *FaG6PDH-CY* promoter can promote the gene expression when plant encountered cold stimuli. Besides, *FaG6PDH-CY* was involved in regulating cold-induced activation of antioxidant enzyme genes (*FaSOD*, *FaCAT*, *FaAPX* and *FaGR*) and RBOH-dependent ROS generation. The elevated *FaG6PDH-CY* enhanced ROS-scavenging capability of antioxidant enzymes to suppress ROS excessive accumulation and relieved the oxidative damage, eventually improving the strawberry resistance to cold stress.

## 1. Introduction

The oxidative pentose phosphate pathway (OPPP) generates nicotinamide adenine dinucleotide phosphate (NADPH) for reductive biosynthesis of metabolic products including fatty acids and amino acids, as well as many intermediary metabolites such as ribulose-5-phosphate for nucleotide synthesis and erythrose-4-phosphate for aromatic amino acids production, which plays an important role in plant life cycle [1]. 

Glucose-6-phosphate dehydrogenase (G6PDH, EC1.1.1.49) is well-known as the first and rate-limiting enzyme of OPPP that catalyzes the conversion of glucose-6-phosphate to 6-phosphogluconolactone and exists widely in animals, plants and prokaryotes [1]. According to their subcellular localization in plant, G6PDHs are divided into cytosolic and plastidic isoforms [2,3]. Cytosolic isoform (Cy-G6PDH) accounts for the major part of total activity of the enzyme in plant cells and generally shows a low sensitivity to reducing power, and its amino acid sequence lacks a transit peptide at the N-end [4,5,6]. Differences in the specific antibodies, biochemical characteristics and gene expression pattern represent a further point for the discrimination of the plastidic isoforms. Two compartmented isoforms in plastids have been defined as P1-G6PDH (also known as chloroplastic G6PDH) that seems to be exclusively found in green tissues, and P2-G6PDH (also known as plastidic G6PDH) that is mainly detected in roots and heterotrophic tissues. P2-G6PDH was less sensitive to NADPH feedback inhibition than P1-G6PDH [7,8]. Subsequently, the basic plastidic but catalytically inactive P0-G6PDH is identified and it can help P1-G6PDH enter peroxisomes [9,10].

In addition to its involvement in plant growth and development, the critical functions of G6PDHs in stress-response mechanisms have been widely documented, such as drought [11], salt [12], cold [13], heat [14], heavy metals [15], nutrient deficiency [16] and disease [17]. In response to various stress, plants have evolved efficient enzymatic and non-enzymatic antioxidative defense systems to suppress accumulation of reactive oxygen species (ROS). G6PDHs provide NADPH for the antioxidant system to protect cells from oxidative damage and maintain cellular homeostasis, thereby enhancing stress resistance in plants [18]. It has been found that overexpression of cytosolic *PsG6PDH* gene from *Populus suaveolens* improves tobacco cold tolerance by supplying sustainable levels of NADPH for SOD and POD scavenging reactions [13]. Additionally, G6PDHs play a central role in the control of output of GSH from its oxidative form (GSSG) by utilizing NADPH, which contribute to enhance ascorbate-glutathione (AsA–GSH) cycle rate and maintain H_2_O_2_ homeostasis under salt stress [19].

Although a major involvement of G6PDHs in stress responses has been widely proven in several plants, little is known about actual biological function of it in fruit trees. Strawberry is a model fruit crop in Rosaceae genomics research and also has high economic and nutritional value. However, strawberry is often subjected to extreme sporadic chilling injury in the short term in early spring and winter as a result of inadequate insulation measures, which causes enormous economic loss. Thus, the goal of the present paper is to investigate the G6PDHs specific role in response to cold stress, which contributes to strawberry breeding for cold resistance.

## 2. Results

### 2.1. Cloning and Characterization of G6PDH Genes in Strawberry

**Figure 1:**
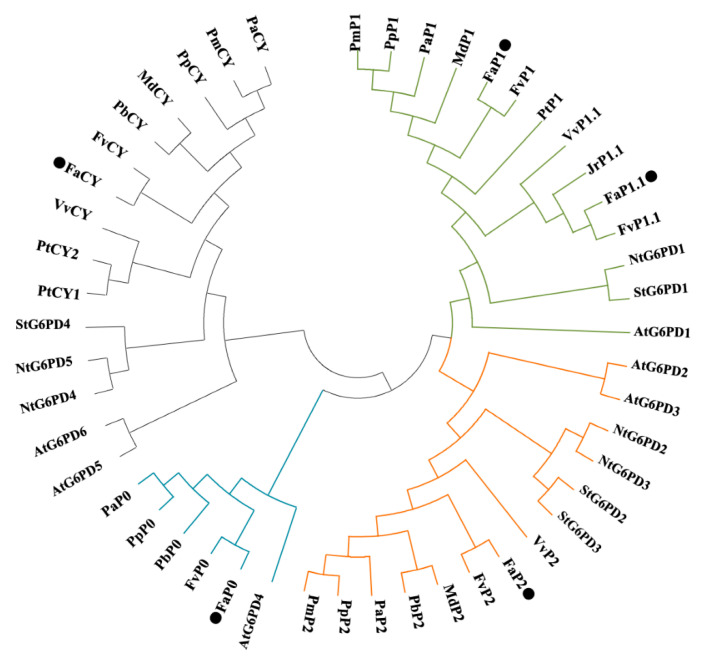
Phylogenetic analysis of G6PDHs from different species. Fa, *Fragaria×ananassa* (black circles); Fv, *Fragaria vesca;* Md, *Malus×domestica*; Pb, *Pyrus×bretschneideri*; Pa, *Prunus avium*; Pp, *Prunus persica*; Pm, *Prunus mume*; Pt, *Prunus tenella*; Vv, *Vitis vinifera*; At, *Arabidopsis thaliana*; Nt, *Nicotiana tabacum*; St, *Solanum tuberosum*; Jr*, Juglans regia.* CY, G6PDH-CY; P1, G6PDH-P1; P2, G6PDH-P2; P0, G6PDH-P0.

Five *FaG6PDH* gene sequences, respectively referred to as *FaG6PDH-CY*, *FaG6PDH-P1*, *FaG6PDH-P1.1*, *FaG6PDH-P2* and *FaG6PDH-P0* were isolated from strawberry (Table S1). The analysis of physicochemical properties showed that the open reading frame (ORF) length of *FaG6PDHs* ranged from 1542 to 1881bp and deduced proteins had 513 to 626 amino acid residues. The protein molecular weight (MW) and theoretical pI varied from 58.5 to 70.8kDa, 5.93 to 8.57, respectively. All FaG6PDH proteins had no signal peptide and transmembrane helix. Besides, FaG6PDH-CY had no transit peptide (Table S2), which can be also confirmed from the multiple sequence alignment that plastidic isoforms were much longer than the FaG6PDH-CY at the N-terminal. Clearly, FaG6PDHs were highly conservative and contained typical domains of the G6PDH family including Rossman fold, substrate-binding site and NADP^+^ binding site (Figure S1). In addition, G6PDHs in strawberry were divided into cytosolic (CY) and plastidic (P1, P2 and P0) types as other species in the phylogenetic tree (Figure 1).

### 2.2. Expression Profile of FaG6PDHs in Different Tissues and during the Fruit Development

**Figure 2:**
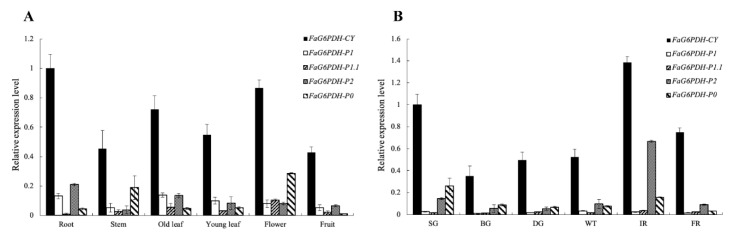
Expression profile of *FaG6PDHs* in different tissues and during the fruit development. (**A**) qRT-PCR analysis of *FaG6PDHs* in different tissues. (**B**) qRT-PCR analysis of *FaG6PDHs* during different fruit developmental stages. SG, small green; BG, big green; DG, de-green; WT, white; IR, initial red; FR, full red.

*FaG6PDHs* constitutively expressed among different tissues, but they displayed variable expression patterns (Figure 2). *FaG6PDH-CY* was the most highly expressed gene among five *FaG6PDH* members and had the highest expression level in root. *FaG6PDH-P1* expressed more highly in leaf, root and flower than stem and fruit. *FaG6PDH-P1.1* transcript kept low-abundance in almost all tissues except flower. *FaG6PDH-P2* had the highest transcript abundance in root, followed by old leaf, and had lowest expression level in stem. *FaG6PDH-P0* had a relatively significant transcript accumulation in flower and stem (Figure 2A). Clearly, *FaG6PDH-CY* expression was remarkably higher than other genes during the fruit development, especially at initial red and small green stages. *FaG6PDH-P1* and *FaG6PDH-P1.1* displayed very low transcript abundance at all stages of fruit development. *FaG6PDH-P2* showed the similar expression pattern to *FaG6PDH-P0*, a relatively higher expression level only at small green and initial red stages (Figure 2B).

### 2.3. Effect of FaG6PDH Enzyme Activity on Related Physiological Indexes in Response to Cold Stress

**Figure 3:**
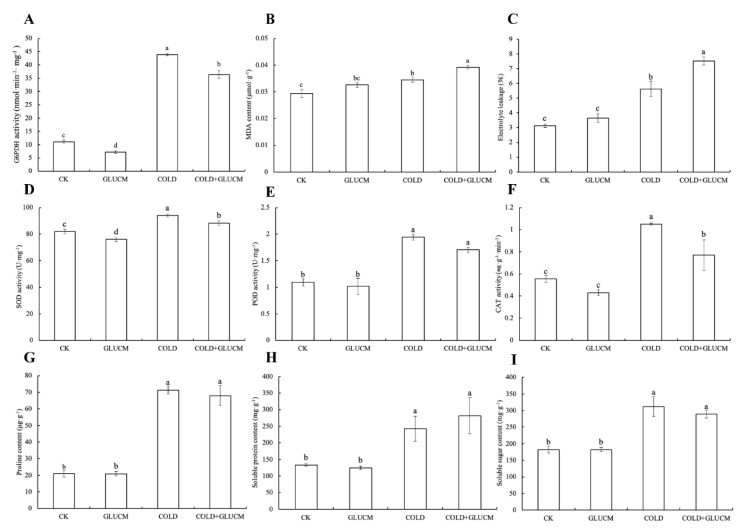
Effect of FaG6PDH enzyme activity on related physiological indexes in response to cold stress. Potted strawberry plantlets respectively treated by distilled water and glucosamine were subjected to room temperature and 0 °C environment for 24 h. Leaves were collected to measure G6PDH activity (**A**), and other related physiological indexes (**B**–**I**). Different lowercase letters indicate significant differences at *p* < 0.05.

The enzymatic assay showed that the FaG6PDH activity significantly increased under cold stress. Using glucosamine (GLUCM, a G6PDH inhibitor) to modulate the FaG6PDH activity, a significant decrease in the FaG6PDH activity was achieved in the control and cold stress (Figure 3A). After the FaG6PDH activity was inhibited, superoxide dismutase (SOD) activity notably decreased, and peroxidase (POD) activity had no significant change, and catalase (CAT) activity evidently decreased only in cold stress (Figure 3D–F). Inhibition of FaG6PDH activity with GLUCM treatment did not affect proline, soluble protein and soluble sugar contents, although these osmoregulation substances were induced by cold stress (Figure 3G–I). Additionally, the malondialdehyde (MDA) content and electrolyte leakage were notably upregulated using GLUCM when strawberry was exposed to cold stress, indicating that plant cell membrane was damaged more seriously when FaG6PDH activity was suppressed under cold stress (Figure 3B,C).

### 2.4. Identification of FaG6PDH Genes Related to Cold Stress

**Figure 4:**
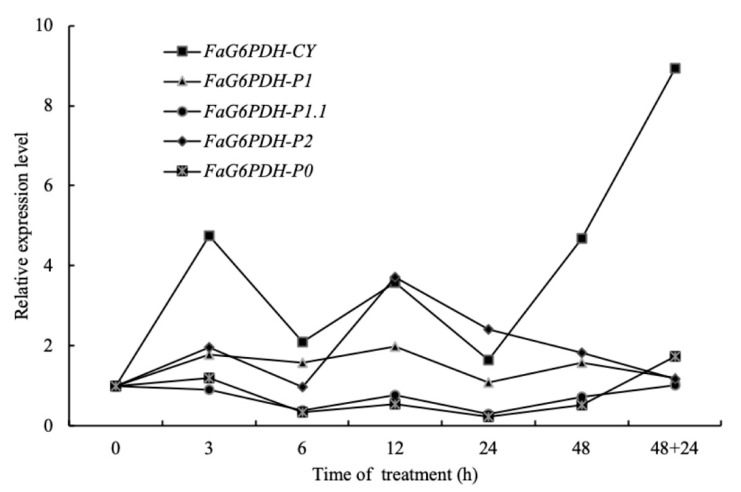
Relative expression level of *FaG6PDH* genes under cold stress. Potted strawberry plantlets were directly subjected to cold stress at 0 °C. Leaves were sampled at 0, 3, 6, 12, 24 and 48 h after treatment. Subsequently, plants were taken out to de-acclimate at room temperature for 24 h. All above samples were used to detect the expression of *FaG6PDH* genes by qRT-PCR.

In order to investigate the sensitivity of five *FaG6PDH* genes to the cold stimuli, transcript abundances of them were detected during the short-term response to cold stress. *FaG6PDH-CY* transcript rapidly accumulated after 3 h cold exposure, and then had a fluctuated trend, and subsequently kept rising and reached the maximum value after 24 h cold de-acclimation. *FaG6PDH-P2* expression level reached the peak value after 12 h cold treatment, but then gradually decreased and eventually had no difference from that without cold stress. *FaG6PDH-P1, FaG6PDH-P1.1* and *FaG6PDH-P0* almost had no dramatic change during the whole process, suggesting that they were not sensitive to cold stress. Overall, *FaG6PDH-CY* probably played an important role in cold tolerance for its significant response to cold stress (Figure 4).

### 2.5. Isolation and Cold Stress-Induced Activity Analysis of FaG6PDH-CY Promoter

**Figure 5:**
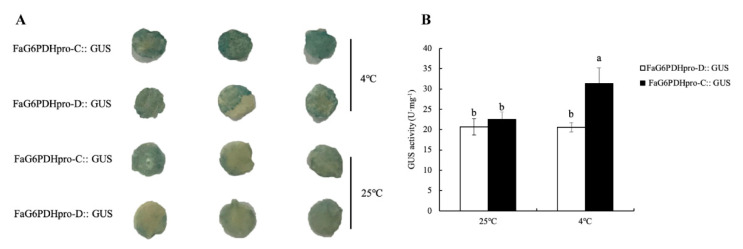
Beta-glucuronidase (GUS) activities in transgenic tobacco leaves under cold stress. FaG6PDHpro-C:: GUS and FaG6PDHpro-D:: GUS seedlings were kept at 4 °C and 25 °C for 48 h. Round leaf discs with a diameter of 1 cm punched from the treated tobacco leaves were performed histochemical staining (**A**) and quantification analysis (**B**) of GUS protein expression. Different lowercase letters indicate significant differences at *p* < 0.05.

1015 bp 5′ flanking sequence of *FaG6PDH-CY* upstream of the ATG start codon from strawberry genomic DNA was isolated. The sequence analysis showed that *FaG6PDH-CY* promoter contained the common cis-acting elements (TATA-box and CAAT-box) and light responsive (Box 4, GT1-motif and P-box), hormone-related (CGTCA-motif, GARE-motif, P-box, TGA-box, TGACG-motif), and stress-related (ARE and LTR) cis-acting regulatory elements (Table S3). The existence of element LTR indicated that *FaG6PDH-CY* was involved in low-temperature responsiveness. Hence, two 5′-deletion fragments of *FaG6PDH-CY* promoter with LTR (FaG6PDHpro-C) or without LTR (FaG6PDHpro-D) were fused to the GUS reporter gene and transferred into tobacco to test the promoter activities under cold stress. As shown in Figure 5, there was a significant increase in the inducible GUS activity of tobacco leaves harboring LTR promoter under cold stress, whereas no significant changes were observed under the normal condition when compared with the control promoter, which indicated that LTR element in *FaG6PDH-CY* promoter can promote high expression of downstream target genes when plant encountered cold signal.

### 2.6. Effect of FaG6PDH-CY Transient Expression on Strawberries in Response to Cold Stress

**Figure 6:**
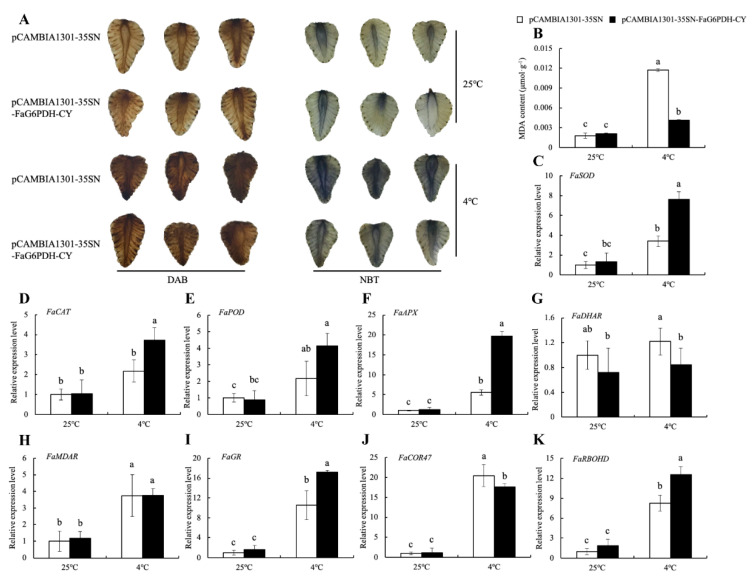
Effect of *FaG6PDH-CY* overexpressing on strawberry fruits in response to cold stress. Overexpressing strawberries were sampled after 5-day treatment at 4 °C and 25 °C to detect ROS production (**A**), MDA content (**B**) and gene expression (**C–K**). H_2_O_2_ was indicated by the presence of deep brown using 3, 3-diaminobenzidine (DAB) staining. O_2_^-^ was indicated by the presence of dark blue using nitrobluetetrazolium (NBT) staining. Different lowercase letters indicate significant differences at *p* < 0.05.

**Figure 7:**
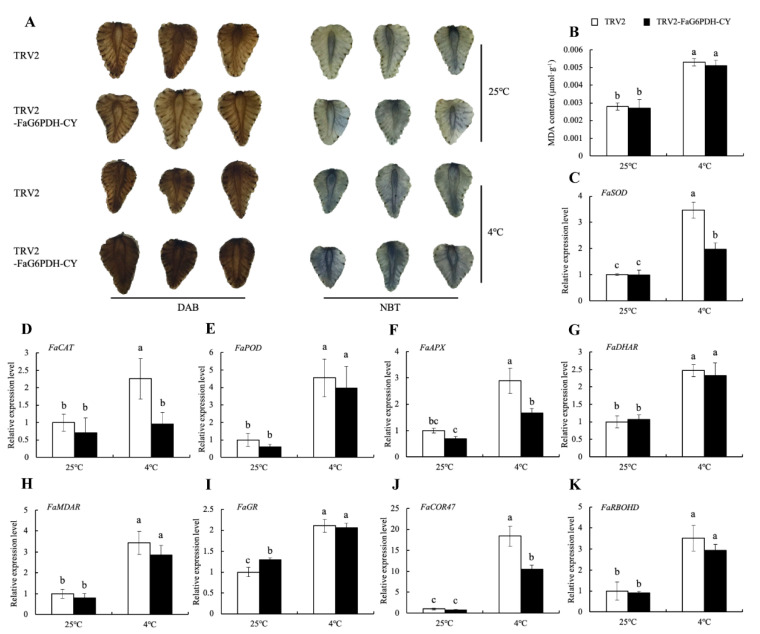
Effect of *FaG6PDH-CY* silencing on strawberry fruits in response to cold stress. silencing strawberries were sampled after 6-day treatment at 4 °C and 25 °C to detect ROS production (**A**), MDA content (**B**) and gene expression (**C–K**). H_2_O_2_ was indicated by the presence of deep brown using 3, 3-diaminobenzidine (DAB) staining. O_2_^-^ was indicated by the presence of dark blue using nitrobluetetrazolium (NBT) staining. Different lowercase letters indicate significant differences at *p* < 0.05.

*FaG6PDH-CY* was transiently expressed in strawberry fruits by overexpressing and silencing strategies to explore its function in response to cold stress (Figures S2 and S3). The DAB and NBT staining showed that cold stress promoted H_2_O_2_ and O_2_^-^ accumulation (Figure 6A and Figure 7A). *FaG6PDH-CY* overexpression had less H_2_O_2_ and O_2_^-^ content than the control under cold stress (Figure 6A), whereas *FaG6PDH-CY* silencing had the opposite effect (Figure 7A). MDA content was significantly reduced in *FaG6PDH-CY* overexpressing fruits compared to the empty vector under cold treatment (Figure 6B), but there were no significant differences between *FaG6PDH-CY* silencing and the control (Figure 7B). In addition, *FaG6PDH-CY* overexpression significantly increased *FaSOD*, *FaCAT*, *FaAPX*, *FaGR* and *FaRBOHD* expression levels in response to cold stress (Figure 6C–K), whereas *FaG6PDH-CY* silencing significantly reduced *FaSOD*, *FaCAT*, *FaAPX* and *FaCOR47* expression levels (Figure 7C–K). These data indicated that alteration of *FaG6PDH-CY* expression affected strawberry cold tolerance.

## 3. Discussion

OPPP is a pivotal source of reducing power and biosynthetic precursors in plants. G6PDH as the key enzyme in the OPPP controls NADPH production and carbon flow, which plays an important role in plant growth, development, and stress responses [20,21,22]. Genes encoding G6PDH, a small gene family have been identified in several plants including potato [5], wheat [23], Arabidopsis [9], barley [24], tomato [11], rubber tree [25] and soybean [26]. Based on the presence of transit peptide, six FaG6PDHs in our study were divided into cytosolic and plastidic two types (Figure 1, Figure S1 and Table S2), which was in accord with previous reports [9,27]. Different G6PDH isoforms distribute in tissue-specific manner. The cytosolic G6PDHs are widespread in all tissues and especially have a high expression in sink tissues, whereas plastidic P1-G6PDHs are predominantly expressed in green tissues and P2-G6PDHs abundance is considerably higher in roots [7,9]. Here, *FaG6PDH-CY* was most abundantly expressed in different tissues and at different fruit developmental stages (Figure 2A,B), indicating that it encoded the major G6PDH isoform throughout the plant and played an important role in various biological processes. It has been reported that cytosolic G6PDHs account for approximately 80% of the total activity of G6PDH in plant tissues [4,28]. *FaG6PDH-P1* and *FaG6PDH-P2* respectively showed highest transcript level in leaves and roots (Figure 2A), consistent with the expression patterns of their orthologues in Arabidopsis, tobacco and rubber tree [9,25,29]. The maximum values of *FaG6PDH-P1.1 and FaG6PDH-P0* were detected in flower (Figure 2A). Additionally, *FaG6PDH-P1* and *FaG6PDH-P1.1* were detectable but their abundances were considerably lower compared with other isoforms during fruit development (Figure 2B). Differential expression of *FaG6PDHs* in distinct tissues suggested that they were probably multifarious players in plant growth and development.

G6PDH activity could be induced by diverse (a)biotic-stress related elicitors. It plays a vital role in maintaining redox balance and protecting cells from oxidative damage by providing the antioxidative defense system with the reducing power [17,30]. FaG6PDH activity was dramatically induced after strawberry was subjected to cold stress, accompanied by significant increasing of SOD, POD, CAT, proline, soluble protein, soluble sugar, MDA and electrolyte leakage. However, activities of antioxidant enzymes decreased, and more MDA and electrolyte leakage produced, when FaG6PDH activity was inhibited by GLUCM, indicating plant suffered greater injury caused by cold stress. This result demonstrated that FaG6PDH positively regulated cold tolerance in strawberry (Figure 3). G6PDH has been shown to respond to various oxidative stresses at the levels of enzyme activity and gene expression [31,32]. Meanwhile, the subcellular location of G6PDH seemed to have certain impacts on their stress responses [26,33]. Quantitative real-time PCR analyzed the expression profile of *FaG6PDH* gene family under cold stress. The result showed that cytosolic *FaG6PDH-CY* expression was significantly upregulated like its enzymatic activity and the plastidic *FaG6PDHs* had no dramatic change, implying a major role of *FaG6PDH-CY* in response to cold stress (Figure 3 and Figure 4). Gene expression is mainly controlled by cis-acting elements in the promoter region that determines many biological process and stress responses [34]. The 1015 bp fragment of *FaG6PDH-CY* promoter contained a putative LTR cis-element (Table S3), indicating that the expression of this gene might be regulated by low temperature, which was confirmed by the cold-inducible increases of GUS activity in the promoter construct containing LTR element (Figure 5).

**Figure 8:**
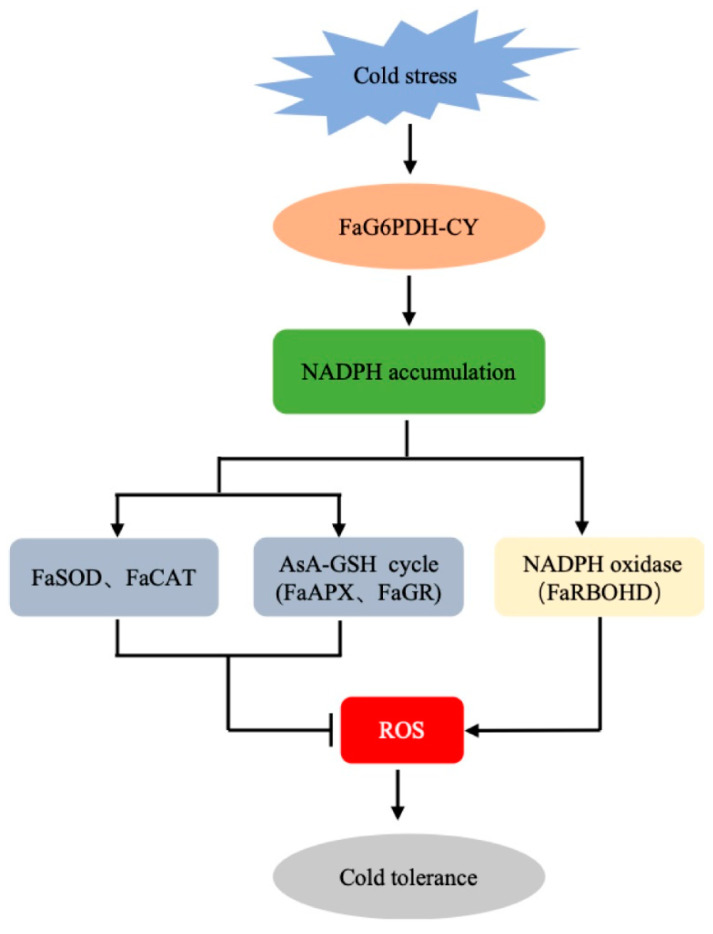
A putative model for FaG6PDH in response to cold stress in strawberry. When plant encountered cold stimuli, FaG6PDH was activated to promote NADPH generation. With the reducing power of NADPH, FaSOD, FaCAT, FaAPX and FaGR scavenged excessive ROS produced by NADPH oxidase (FaRBOHD) and protected cells from oxidative damage, thereby conferring the cold resistance to plant.

To further understand the role of *FaG6PDH-CY* gene in cold-stress response, its functionality has been evaluated by transient expression assays. The burst out of ROS is one of the common phenomena in plant in response to different biotic and abiotic stresses [35]. In our study, cold stress promoted ROS production (Figure 6A and Figure 7A). It has been reported that cytosolic G6PDHs participate in the modulation of cellular redox states by supplying NADPH [26,36]. We found that the levels of ROS were obviously lower in *FaG6PDH-CY* overexpression than control under cold stress, and the cellular membrane damage in cold-exposed overexpression strawberry was less severe as manifested by the notable decrease in MDA content (Figure 6B). These evidences showed that *FaG6PDH-CY* overexpression could inhibit the cold-induced ROS accumulation, and consequently alleviate the membrane lipid peroxidation and confer cold tolerance. On the contrary, silencing *FaG6PDH-CY* enhanced ROS generation under cold stress, but it did not significantly trigger cellular membrane damage (Figure 7A,B), which probably resulted from incomplete silencing of *FaG6PDH-CY* or indicated that *FaG6PDH-CY* would be an indirect factor in the development of cold tolerance. SOD, CAT, POD, and those enzymes (APX, DHAR, MDAR and GR) implicated in the ascorbate-glutathione (AsA–GSH) cycle are important ROS scavengers, with NADPH required as a reducing agent in these scavenging reactions [37,38]. Exposure to cold stress, *FaG6PDH-CY* overexpression significantly up-regulated *FaSOD* and *FaCAT* expression levels (Figure 6C,D), whereas *FaG6PDH-CY* silencing significantly down-regulated their expression levels (Figure 7C,D), which was consistent with change of enzymatic activity (Figure 3D,F). Besides, *FaG6PDH-CY* modulated AsA–GSH pathway to resist cold stress by predominantly affecting *FaAPX* and *FaGR* transcript levels (Figure 6F,I and Figure 7F,I), in agreement with the research in salt-induced cy-G6PD gene function in Arabidopsis [39]. Cold-responsive gene *COR47* was reported to involve the strawberry cold acclimation [40]. However, our data suggested that it was not regulated by *FaG6PDH-CY* expression under cold stress (Figure 6J and Figure 7J). NADPH oxidases, also named respiratory burst oxidase homologues (RBOHs), require NADPH to generate the superoxide anion (O_2_^-^), subsequently ending with the secondary production of other ROS such as hydrogen peroxide (H_2_O_2_) [41]. Our previous study demonstrated that RBOHD was important in response to cold stress in strawberry [42]. As shown in Figure 6K and Figure 7K, the expression of *FaRBOHD* was markedly increased under cold treatment, and significantly higher in overexpressing *FaG6PDH-CY* than that in control. In contrast, *FaRBOHD* expression level was reduced in silencing *FaG6PDH-CY* compared to control. These results indicated that *FaG6PDH-CY* was involved in RBOH-dependent ROS production in cold-stressed strawberry. Taken together, function of *FaG6PDH-CY* in response to cold stress is achieved through *FaSOD, FaCAT*, *FaAPX*, *FaGR* and *FaRBOHD*. On the basis of our above results and previous reports, we propose the model shown in Figure 8. In cold stress, *FaG6PDH-CY* was induced and produced more NADPH for a defense response, which enhanced antioxidant ability that contributed to scavenge excessive ROS generated by NADPH oxidase, and eventually alleviated the cold-induced oxidative damages.

## 4. Materials and Methods

### 4.1. Plant Materials and Treatments

Strawberries (*Fragaria×ananassa* cv. Benihoppe) are grown in a simple greenhouse at Chengdu campus of Sichuan Agricultural University (30°42’N, 103°51´E) with the routine management. Strawberry root, stem, young leaf, old leaf, flower and fruit (small green, big green, de-green, white, initial red and full red) were collected for tissue- and stage-specific transcript analysis of *G6PDH* genes. Potted strawberry plantlets with vigorous and uniform growth status were selected for the cold treatments. One set of plantlets was treated with 100 mM glucosamine (GLUCM, a G6PDH inhibitor) by spraying evenly over the whole leaf. Another set of plantlets as control was treated with distilled water. Half of the above treated plantlets were kept at room temperature, and the others were put into 0 °C environment. Leaves were collected after 24 h. In addition, some potted strawberries were directly subjected to cold stress at 0 °C, followed by sampling at 0, 3, 6, 12, 24 and 48 h after treatment. Subsequently, plants were taken out to de-acclimate at room temperature for 24 h. *Nicotiana benthamiana* seeds were sowed in the soil (peat, perlite and vermiculite, 2:1:1 in volume ratio) and cultured in the growth room (23 ± 2 °C, 16 h light/8 h dark, 6000 l×). The strawberry fruits for transient expression were harvested from a local farm at Shuangliu County, Sichuan Province (southwest China).

### 4.2. Determination of G6PDH, SOD, CAT and POD Activities

The extract was prepared for G6PDH activity and assayed using the commercial kits purchased from SuZhou Keming Bioengineer Company (SuZhou, China), following the manufacturer’s instruction. G6PDH can reduce NADP^+^ to NAPDH in OPPP reaction. The G6PDH activity was measured by increasing rate of NAPDH at 340 nm. One unit (U) of enzyme activity was defined as the amount of enzyme that produced 1 nmol of NAPDH per milligram of protein in 1 min. A total of 0.2 g samples was homogenized in 10 mL ice-cold potassium phosphate buffer (50 mM, pH 7.8) and then centrifuged at 8000 r/min for 20 min at 4 °C. The supernatant was prepared for assay of SOD, CAT and POD activities. All of them were measured according to the protocols described by Xiong (2003) [43]. SOD activity was detected by monitoring its ability to inhibit the photochemical reduction of NBT. CAT activity was determined using potassium permanganate titration. POD activity was evaluated with guaiacol colorimetric method.

### 4.3. Electrolyte Leakage and MDA Content Assay

Electrolyte leakage and MDA content were assayed based on the methods described by Xiong (2003) [43]. Leaves were washed and immersed in de-ionized water for 2 h at 25 °C. The conductivity in the solution was measured (C1). Samples were then incubated in boiling water for 1 h and cooled down to the room temperature before the ultimate conductivity was measured (C2). Relative electrolyte leakage was expressed as the ratio C1/C2. A total of 0.3 g samples was homogenized in 8 mL of 10% (*w*/*v*) trichloroacetic acid (TCA). The homogenate was centrifuged at 5000 r/min for 10 min. MDA content in the supernatant was determined using the thiobarbituric acid method.

### 4.4. Proline, Soluble Sugar and Soluble Protein Content Determination

Finely ground leaf tissue (0.2 g) was suspended in 5 mL of 3% sulphosalicylic acid and then centrifuged at 5000 r/min for 10 min. The proline content in the solution was determined by acid ninhydrin method [43]. A total of 0.5 g samples was ground and added into 8 mL distilled water. The homogenate was centrifuged at 5000 r/min for 10 min. The supernatant was used for soluble sugar and soluble protein determination. The content of soluble sugar was measured using anthrone colorimetric method and the soluble protein was quantified by Coomassie brilliant blue G-250 [43].

### 4.5. RNA Extraction, First-Strand cDNA Synthesis and Genomic DNA Isolation

Total RNA and genomic DNA were isolated from tissue samples according to the protocols described by Chen et al. (2012) [44]. After quantity and quality evaluation, 1 µg of total RNA was reversely transcribed into the first-strand cDNA using PrimeScript^TM^ RT Reagent Kit with gDNA Eraser (Perfect Real Time) (Takara, Japan) according to the manufacturer’s instructions.

### 4.6. Cloning and Bioinformatic Analysis of G6PDH Genes

Five strawberry G6PDH sequences were downloaded using Arabidopsis G6PDHs as query probes to BLAST search against GDR (Genome Database for Rosaceae) and NCBI (National Center for Biotechnology Information) databases. Except for *FaG6PDH-CY* (KC433888), *FaG6PDH-P1* (KC433889), *FaG6PDH-P2* (JQ260862.1) submitted to NCBI in our previous study, another two members *FaG6PDH-P1.1* and *FaG6PDH-P0* were cloned using primers (*FaG6PDH-P1.1*: 5′-GTCTACACGATTTAGCCGACATGG-3′, 5′-GCTTACACCTGACAATGGATGAGTG-3′; *FaG6PDH-P0*: 5′-AAGATCTTAGACCTGAACAGGGA-3′, 5′-AGAGTAGCCGTTGTACCTCACAA-3′). Subsequently, all strawberry G6PDHs were performed bioinformatic analysis using ExPASy-ProtParam for physicochemical parameter computation, TargetP 2.0 and ProtComp 9.0 for N-terminal transit peptide (TP) prediction, SignalP 4.1 for signal peptide (SignalP) prediction, TMHMM 2.0 for transmembrane helix (TMH) prediction, DNAMAN for multiple sequence alignment, ClustalX and MEGA for phylogenetic tree construction. Additionally, to obtain the *FaG6PDH-CY* promoter, approximately 1000 bp upstream sequence flanking start codon was amplified with specific primers (5′-CTGCTTGACTTGGTGCTGAAAAC-3′ and 5′-CCTTCCTGAGCTGTTGCGC-3′) using genomic DNA. Putative cis-acting elements of *FaG6PDH-CY* promoter were identified by PlantCare online tool.

### 4.7. Quantitative Real-Time PCR

qRT-PCR was conducted using SYBR Green Premix Ex Taq ^TM^ (Takara, Japan) on the CFX96 real-time PCR system (Bio-Rad, Hercules, CA, USA). The 20 μL reaction mixture included 10 μL SYBR Premix, 0.8 μL each primer (0.4 μM), 1.5 μL cDNA template and 6.9 μL ddH_2_O. Reaction protocol was set as follows: 95 °C for 3 min, followed by 40 cycles of 95 °C for 10 s, 55 °C for 30 s and 72 °C for 15 s. After that, melting curve was inserted to verify specificity of primer amplification. The relative expression level was calculated with the 2 ^−^^△^^△Ct^ method. Primers designed for the target and internal control genes were listed in Table S4.

### 4.8. Vector Construction

For functional validation of *FaG6PDH-CY* promoter in response to cold stimuli, two 5′-deletion fragments of the promoter, 393 bp FaG6PDHpro-C with LTR element and 323 bp FaG6PDHpro-D without LTR element were amplified with primers (FaG6PDHpro-C: 5′-CCAAGCTTCCGAAATTCGATCGCGCTCT-3′, 5′-GCTCTAGACCTTCCTGAGCTGTTGCGCA-3′; FaG6PDHpro-D: 5′-CCAAGCTTTTCGATCGCGCTCTGCTCAAT-3′, 5′-GCTCTAGACCTTCCTGAGCTGTTGCGCA-3′). The obtained amplicons were used to replace the CaMV 35S promoter in pBI121 vector with help of *Hin*dⅠⅠⅠ and *Xba*Ⅰ (Figure S4). FaG6PDHpro-C:: GUS and FaG6PDHpro-D:: GUS plasmids were prepared for tobacco transient transformation assay. For overexpression, the coding sequence of *FaG6PDH-CY* gene was amplified with primers (o*FaG6PDH-CY*: 5′-GGGGTACCGAGCTACGTAGTTGCGCAACAG-3′, 5′-GCTCTAGAACACACAATACCGCACTC-3′) and cloned into the modified pCAMBIA1301 vector driven by CaMV 35S promoter using restriction enzymes *Kpn*Ⅰ and *Xba*Ⅰ. For virus-induced gene silencing (VIGS), the tobacco rattle virus RNA1 (pTRV1) and tobacco rattle virus RNA2 (pTRV2) vectors were applied. A 680 bp fragment specifically targeting *FaG6PDH-CY* gene was amplified with primers (v*FaG6PDH-CY*: 5′-GGGGTACCATATCTTATCCAAGGAGCTTCCTCCA-3′, 5′-GCTCTAGATCGTCGTTAATTGGAAGTACCGATT-3′) and then subcloned into pTRV2 with *Kpn*Ⅰ and *Xba*Ⅰ.

### 4.9. Transient Expression and Cold-Resistance Assay in Tobacco Leaves and Strawberry Fruits

Agrobacterium-mediated transient expression assays were performed. All recombinant and empty vectors were introduced into the agrobacterium strain GV3101 by freeze-thaw method. The pelleted cells were resuspended in infiltration medium (10 mM MgCl_2,_ 10 mM MES pH 5.6, 200 μM AS). The agrobacterium suspensions (OD600 = 0.4) harboring FaG6PDHpro-C:: GUS and FaG6PDHpro-D:: GUS vectors were respectively injected into the abaxial surfaces of the leaves of 30 day-old tobaccos using a 1 mL needleless syringe. After 24 h cultivation in dark, the transgenic tobacco plants were transferred to 4 °C and kept for 48 h. The cell suspensions containing pCAMBIA1301-G6PDH-CY and pCAMBIA1301-35SN were respectively diluted to OD600  =  0.8–1.0 using infiltration medium. In addition, the OD600 of TRV1, TRV2 and pTRV2-G6PDH-CY cultures were regulated to 1.6–2.0. Subsequently, Equal volumes of TRV1 and TRV2/ TRV2-G6PDH-CY cultures were mixed together. Transient overexpression and VIGS by infiltration of corresponding construct into strawberry fruits followed the procedures described by Chen et al. (2018) [45]. The treated strawberries were cultured in dark for 12 h, and then subjected to 4 °C treatment. The overexpressing samples were harvest after 5 days and VIGS fruits were collected after 6 days.

### 4.10. Histochemical Staining and Activity Assays for GUS

Round leaf discs with a diameter of 1 cm were punched from the treated tobacco leaves using a hole puncher and then stained by a GUS staining kit (Solarbio, Beijing). A total of 0.2 g treated samples was homogenized in 1.8 mL ice-cold potassium phosphate buffer (50 mM, pH 7.8) and then centrifuged at 8000 r/min for 20 min at 4 °C. The aqueous (upper) phase was analyzed by ELISA kit (Mlbio, Shanghai) for GUS activity.

### 4.11. DAB and NBT Staining

The treated fruits were cut into discs with a razor blade and immersed them in 0.1% (*w*/*v*) DAB or 0.1% (*w*/*v*) NBT staining solution for detection of H_2_O_2_ or O_2_^-^, respectively. The tubes were wrapped with aluminum foil and kept into the 80 r/min shaker at 25 °C for 4–6 h. Then, samples were put into absolute ethanol and heated in a boiling water-bath for 20 min or more (intermittent refreshing of absolute ethanol) for proper visualization of the staining. After that, the residual alcohol on fruit surface was removed by absorbent paper. The change in fruit color was observed and photographed.

### 4.12. Statistical Analyses

All data presented here are mean± standard error of at least three independent biological replicates. The statistical significance of the difference was evaluated by Data Processing System (DPS) using Duncan’s multiple range test at *p* < 0.05.

## Supplementary Materials

The following are available online at https://www.mdpi.com/1422-0067/21/19/7322/s1. Table S1. Sequences of G6PDH family in strawberry; Table S2. Physicochemical properties of strawberry *G6PDH* gene family; Table S3. The cis-acting regulatory elements of *FaG6PDH-CY* promoter; Table S4. Primers for quantitative real-time PCR; Figure S1. Multiple alignment of protein sequences of strawberry G6PDHs; Figure S2. Transient overexpression of *FaG6PDH-CY* in strawberry fruit; Figure S3. Transient silencing of *FaG6PDH-CY* in strawberry fruit; Figure S4. Schematic diagram of constructs used in tobacco transient transformation assay.
