# Supplementary material for: Identification of the Cytosolic Glucose-6-Phosphate Dehydrogenase Gene from Strawberry Involved in Cold Stress Response
Source: Int J Mol Sci. 2020 Oct 3;21(19):7322. doi: 10.3390/ijms21197322 (PMC7582851; doi:10.3390/ijms21197322)
Supplement: Supplementary file 1 [file ijms-21-07322-s001.zip › Supplementary File/Figure S1-S4 .docx]

**
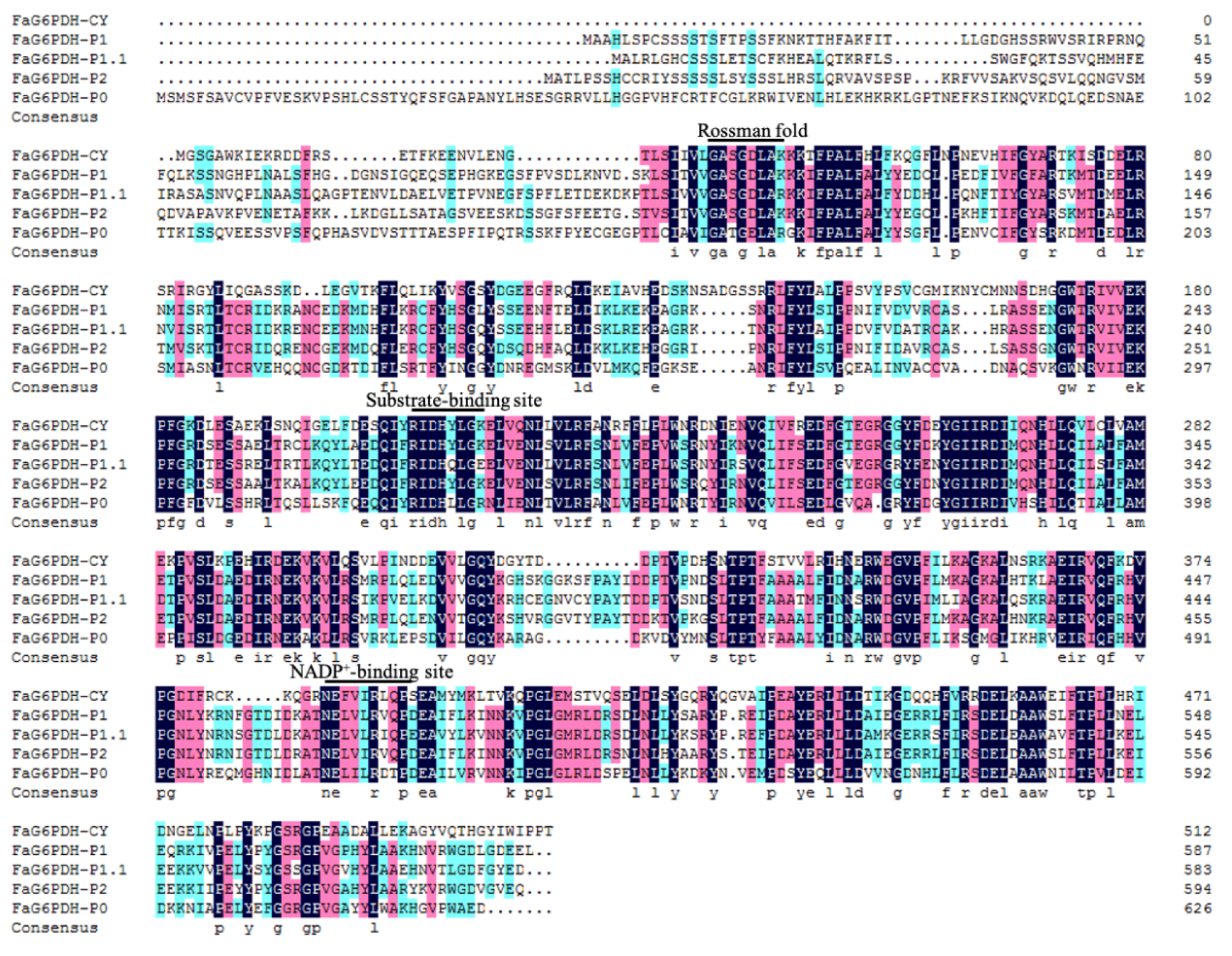
**

**Figure S1. Multiple alignment of protein sequences of strawberry G6PDHs.** Conservative residues are highlighted by black shadings, and a lower level of conservations is represented by lighter shadings. Rossman fold, substrate-binding site and NADP^+^ binding site are indicated by straight lines.

**
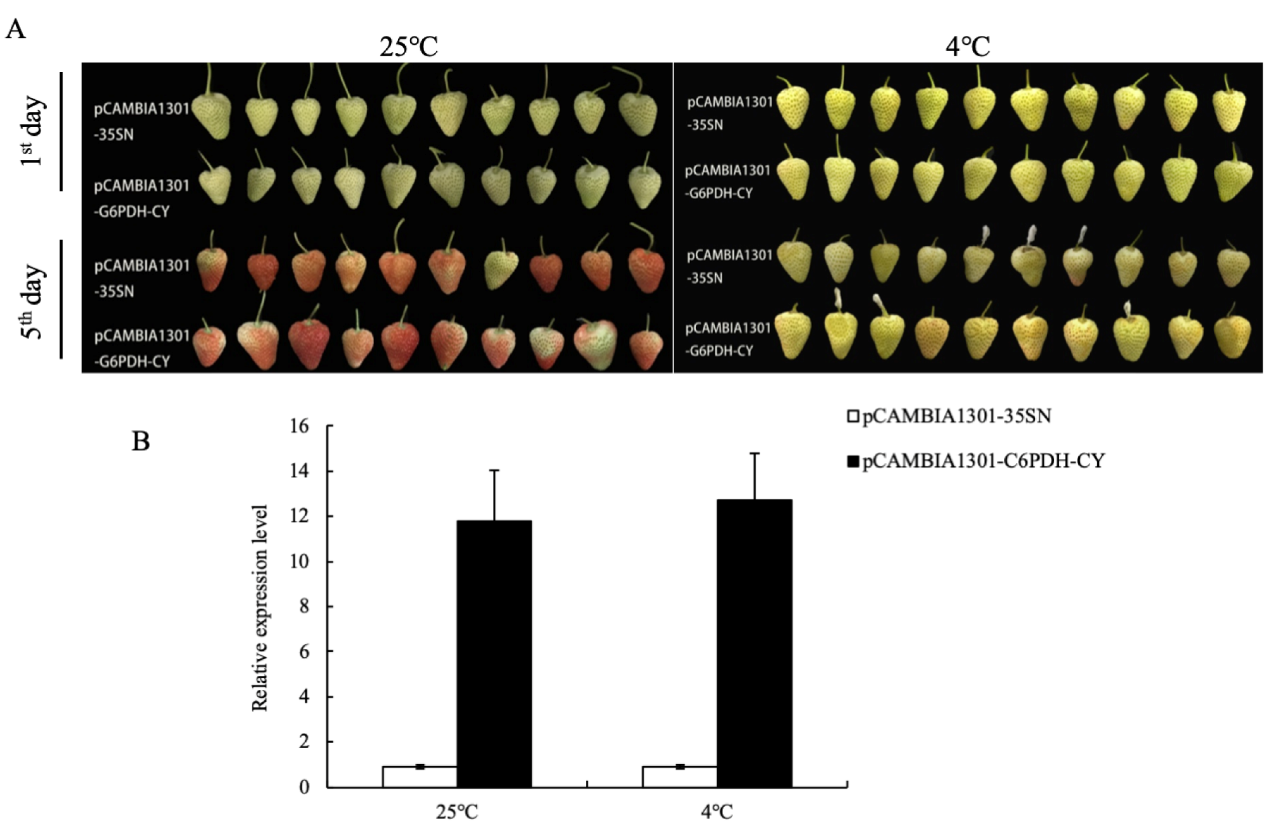
**

**Figure S2. Transient overexpression of *FaG6PDH-CY* in strawberry fruit.** (A) Strawberry fruit phenotypes after *FaG6PDH-CY* overexpressing. (B) *FaG6PDH-CY* relative expression level in the 5^th^ day overexpressed fruits.

**
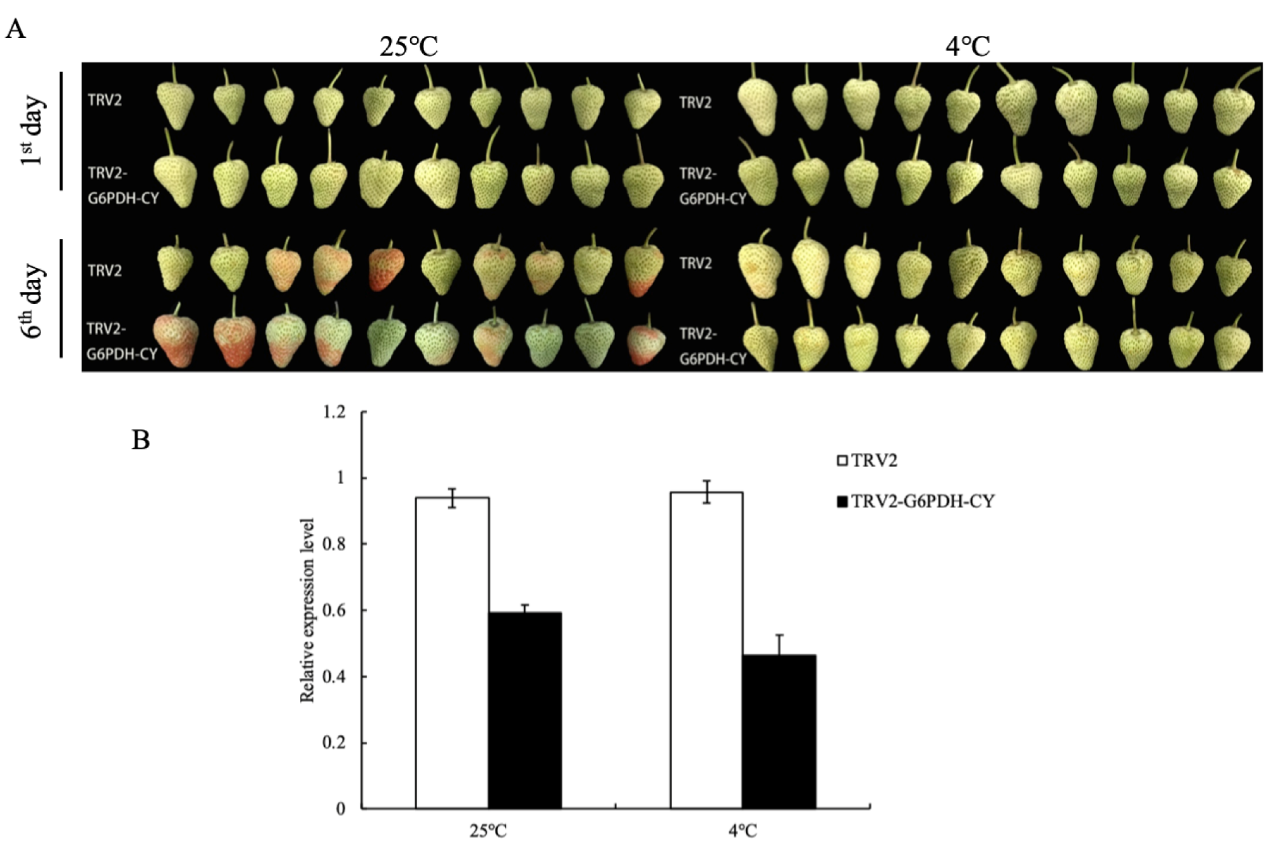
**

**Figure S3. Transient silencing of *FaG6PDH-CY* in strawberry fruit.**  (A) Strawberry fruit phenotypes after *FaG6PDH-CY* silencing. (B) *FaG6PDH-CY* relative expression level in the 6^th^ day silenced fruits.

**
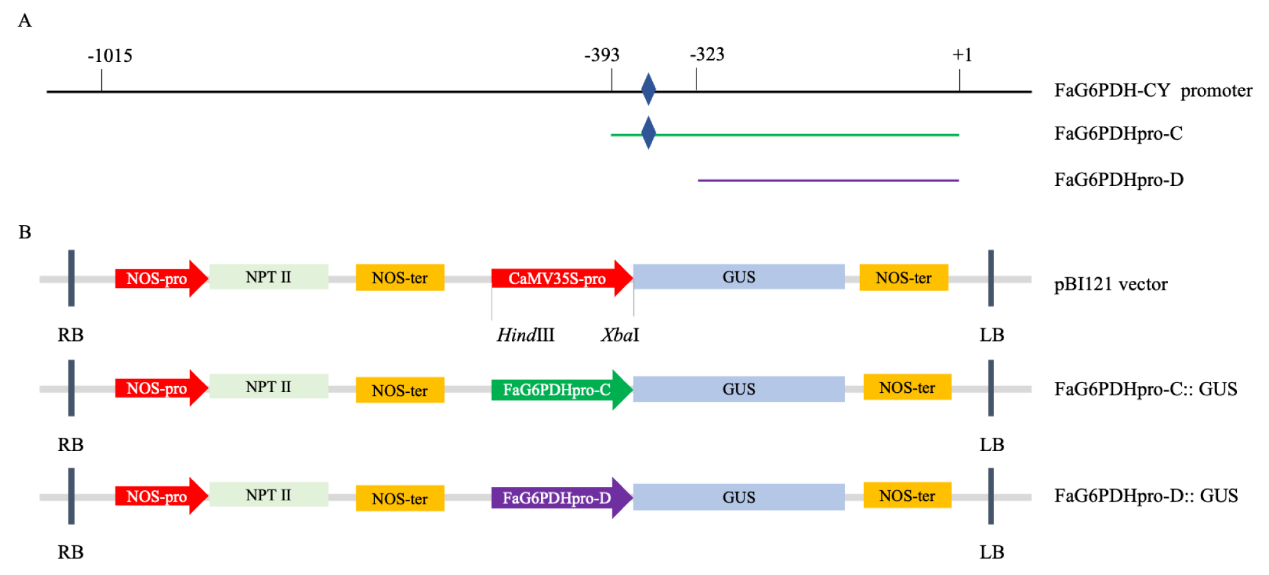
**

**Figure S4. Schematic diagram of constructs used in tobacco transient transformation assay.** (A) 5′-deletion fragments of the *FaG6PDH-CY* promoter. The diamond pattern represents the LTR element. (B) FaG6PDHpro:: GUS vector constructs. These constructs are based on the pBI121vector. The insertion position of 5′-deletion fragments in the vector is indicated with restriction enzyme sites (*Hin*dIII and *Xba*Ⅰ). Nos-ter, nopaline synthase terminator; GUS, β- glucosidase gene; CaMV35S-pro, cauliflower mosaic virus 35S promoter; NPT II, neomycin phosphotransferase II gene; RB and LB, left and right T-DNA borders.
